# Risk factors for drug therapy problems among Cambodian Americans with complex needs: a cross-sectional, observational study

**DOI:** 10.1080/21642850.2021.2021917

**Published:** 2022-01-24

**Authors:** Julie A. Wagner, Angela Bermudez-Millan, S. Megan Berthold, Thomas Buckley, Orfeu M. Buxton, Richard Feinn, Theanvy Kuoch, Sengly Kong, Mackenzie Lim, Christina Polomoff, Mary Scully

**Affiliations:** a Behavioral Sciences and Community Health, University of Connecticut School of Dental Medicine, Farmington, CT, USA; b Public Health Sciences, University of Connecticut School of Medicine, Farmington, CT, USA; c University of Connecticut School of Social Work, Hartford, CT, USA; d Department of Pharmacy Practice, University of Connecticut School of Pharmacy, Storrs, CT, USA; e Department of Biobehavioral Health, Pennsylvania State University, University Park, PA, USA; f Department of Medical Sciences, Quinnipiac University School of Medicine, Hamden, CT, USA; g Khmer Health Advocates, West Hartford, CT, USA

**Keywords:** Drug therapy problem, depression, risk factors, Cambodian, Community health workers‌

## Abstract

**Background:**

Pharmaceutical drug therapy problems (DTPs) are a major public health problem. We examined patient-level risk factors for DTPs among Cambodian Americans.

**Methods:**

Community health workers (CHWs) verbally administered surveys and completed a detailed medication review form with participants. A doctoral-level pharmacist reviewed the form with the patient and CHW to determine DTP number and type (appropriateness, effectiveness, safety, and adherence).

**Results:**

Participants (*n* = 63) averaged 55 years old, 6 years of education, 52% were married, 87% spoke Khmer at home, with modal household income <$20,000 (41%). The percentage of participants with DTPs was: 45% appropriateness, 25% effectiveness, 64% safety, and 30% adherence, averaging 3.7 DTPs per patient. In multiple regressions, patient characteristics uniquely predicted each type of DTP. In a multiple regression controlling for number of medications, being married reduced total DTPs (IRR = 0.70) and being depressed increased total DTPs (IRR = 1.26).

**Conclusions:**

Vulnerable patients should be prioritized for pharmacist/CHW teams to identify DTPs.

**Trial registration:** ClinicalTrials.gov identifier: NCT02502929.

## Introduction

A drug therapy problem (DTP) is an unwanted actual or potential incident related to medication therapy that adversely affects the desired goals of treatment (Niriayo, Kumela, Kassa, & Angamo, 2018). When a medication is not given or taken appropriately, safely, or as intended, it creates a DTP (Patel, Campbell, Moslem, Spriggel, & Warholak, 2020). DTPs can lead to adverse drug events, which can harm patients and incur healthcare costs. DTPs are common and without intervention, they can be persistent over time (Roth, Esserman, Ivey, & Weinberger, 2011). DTPs are the 14th leading cause of morbidity and mortality in the world, putting patient harm from medications in the same league as tuberculosis and malaria (Jha et al., 2013). The World Health Organization has identified DTPs as a global priority and recommends a person-centered approach to avoid harm (World Health Organization, 2019). Thus, identifying the characteristics of patients who are most likely to experience DTPs is critical.

DTPs may be difficult to assess in linguistic and cultural minorities. Recent literature promotes the use of pharmacist/community health worker teams (Segal, Angaran, Odedina, Zeigler, & Wallace, 2020; Wheat et al., 2020). This study employed teams of pharmacists and bilingual, bicultural community health workers to examine DTPs in a high-risk population, i.e., Cambodian Americans with depression and an elevated risk for diabetes.

The literature on risk factors for DTPs overwhelmingly focuses on the characteristics of the medication *regimen*. Consistent findings are that worse health and higher drug count, i.e. polypharmacy, are associated with higher DTPs, presumably because more health problems and medications to treat them increase the opportunity for DTPs (Degu, Njogu, Weru, & Karimi, 2017; Eassey, Smith, Krass, Mc, & Brien, 2016; Lee, Yang, Stockl, Lew, & Solow, 2016; Njeri, Ogallo, Nyamu, Opanga, & Birichi, 2018; Snyder, Frail, Jaynes, Pater, & Zillich, 2014). Far less is known about psychosocial and contextual patient characteristics that increase the risk for DTPs. Demographically, older individuals (Niriayo et al., 2018), those with lower income, and racial/ethnic minorities have higher DTPs (Lee et al., 2016; Roth, Esserman, Ivey, & Weinberger, 2010). Social support may also be important. For example, one study found that patients recently discharged from the hospital for cardiovascular problems were at higher risk of DTPs if they lacked a caregiver at home (Aggarwal, Liao, & Mosca, 2013). Poor patient understanding of their condition and the medications to treat it may be critical (Eassey et al., 2016). Elevated symptoms of depression have also been linked to DTPs (Xiong, Li, Mao, & Xu, 2014).

Cambodian Americans arrived in the U.S. as refugees in the 1980s fleeing the genocidal Pol Pot regime (Wagner et al., 2015). Many are socially isolated (Berthold et al., 2019) and suffer from chronic diseases including diabetes (Marshall et al., 2016), poor self-reported health and physical functioning (Wagner et al., 2013), and serious and persistent mental health sequelae (Marshall, Schell, Elliott, Berthold, & Chun, 2005). Although most have health insurance (Berthold et al., 2014), the quality of their healthcare and their active involvement in their own care may be compromised by low educational attainment (Wagner et al., 2015), low English fluency (Berthold et al., 2014), as well as by structural barriers to quality care (Wong et al., 2006). Many of these patients encounter frequent problems with medications (Buckley, Kuoch, & Scully, 2019) and, in some cases, may receive inadequate mental health treatment (Wong, Marshall, Schell, Berthold, & Hambarsoomians, 2015).

Gathering data from such a population is challenging due to a number of factors, including language, cultural differences in conceptualization of health and medications, deference to healthcare providers and a reluctance to voice disagreement, and psychiatric symptoms that may interfere with symptom reporting. Community health workers (CHWs) are well-positioned to overcome these challenges to provide the pharmacist with a fuller understanding of patient characteristics.

Most investigations of risk factors for DTPs focus on characteristics of the medication regimen. Strategies to identify patients who would benefit from pharmacist-delivered medication therapy management (MTM) to correct DTPs are limited by a lack of literature regarding patient-level risk factors for DTPs. The purpose of this study was to employ bilingual, bicultural CHWs specifically trained to work with pharmacists, to examine patient-level psychosocial and contextual risk factors for DTPs among Cambodian Americans. We hypothesized that patient characteristics would predict number of DTPs above and beyond total number of medications.

## Methods

### Design

This was a cross-sectional, observational study. STROBE guidelines (von Elm et al., 2007) were followed for reporting. Secondary data analysis was conducted on baseline assessments from a diabetes prevention trial for Cambodian Americans with depression, the Diabetes Risk Reduction through Eat, Walk, Sleep and Medication Therapy Management (DREAM) trial (Wagner et al., 2021), clinicaltrials.gov identifier NCT02502929. Previously published articles provide details of the methods and a CONSORT diagram (Polomoff et al., 2021; Wagner et al., 2021). The data reported here are from baseline assessments only and solely for those participants who were randomized to receive MTM intervention and therefore completed a DTP review.

### Participants and sampling

Participants were recruited through referrals from clinics and social service agencies; outreach at large cultural gatherings such as Cambodian new year celebrations; posters at local Khmer businesses; and recruitment events at relevant temples and churches. Inclusion criteria were: (1) aged 35–75; (2) Cambodian or Cambodian-American; (3) Khmer speaking; (4) currently living in Connecticut, Massachusetts, or Rhode Island (northeastern U.S.); (5) lived in Cambodia during or in the immediate aftermath of the Pol Pot regime (1975–1979); (6) ambulatory; (7) consumed meals by mouth; (8) elevated risk for diabetes per the American Diabetes Association Risk Test (American Diabetes Association) modified for this population (International Diabetes Federation, 2005; van Abeelen et al., 2012). Participants were also required to meet criteria for depression by (a) current antidepressant medication and/or (b) elevated depressive symptoms indicative of likely major depressive disorder on the Khmer language Hopkins Symptom Checklist (Mollica, Wyshak, de Marneffe, Khuon, & Lavelle, 1987) with elevated symptoms on two occasions that were two weeks apart during a study screening and eligibility period. Exclusion criteria were: type 2 diabetes; seeing or hearing problems that would interfere with group sessions; major medical problems requiring intensive treatment; pregnancy or planning pregnancy; serious thinking or memory problems (e.g. schizophrenia or dementia); and 3 or more days in a psychiatric hospital or self-harm in the past 2 years. Data were collected from March 2016 to March 2019.

### Community health workers and pharmacists

The five CHWs were born in Cambodia, bilingual and bicultural. CHWs met with participants to complete a medication review form. Their training to decrease bias during survey administration and MTM has been previously described (Polomoff et al., 2021; Wagner et al., 2021). Training the CHWs included shadowing expert pharmacists complete the medication review form, sessions with our pharmacist investigator and the bilingual study coordinator totaling 4 h, and role play with illustrative case examples. CHWs were supervised live for at least 10 medication review sessions with participants and judged by the study coordinator to be satisfactory prior to working independently. Training materials included several online videos, a 34-page toolkit for reading medication labels, a guide to commonly prescribed medications, and a 14-page manual for conducting medication reviews.

Four pharmacists conducted MTM. All were either nationally certified in MTM or board-certified through Board of Pharmacy Specialties in ambulatory care, geriatric, or psychiatric pharmacy and were experienced in providing comprehensive MTM, which inherently includes the assessment of DTPs.

### Procedures

This study was conducted in accordance with the principles stated in the Declaration of Helsinki. The UConn Health institutional review board approved all participant procedures. Participants signed written informed consent forms in their preferred language (Khmer or English). Baseline assessments were then conducted, including surveys, bloodwork, and anthropometrics. Participants were paid $30 USD each in gift cards to a local pharmacy for completing the entire study assessment. Following baseline assessments, participants were randomized; due to resource constraints, only those who were randomized to the MTM intervention had a DTP review.

Participants randomized to MTM met with the CHW in a face-to-face session to create a comprehensive medication record that included medication allergies and reactions, tobacco/alcohol/betel nut use, medication name/dose/directions, how the participant is actually taking the medication, participant satisfaction with medication(s), prescriber and pharmacy names and phone numbers. The CHW collected information regarding all prescription and over-the-counter medications, herbal products, dietary supplements, and traditional Khmer medicines.

Next, the participant, CHW, and pharmacist met together. The pharmacist was in his/her office and videoconferenced with the participant and CHW who were together face-to-face in their locale. Pharmacists reviewed the medication list and medication use history form. Relevant data from the research record were available to the pharmacist, including blood assays (i.e. HbA1c and lipid profile), physical assessment findings (i.e. body mass index and blood pressure), medication-related patient surveys (i.e. beliefs about medicine), and psychosocial and lifestyle information (i.e. depressive symptoms, pain, self-reported sleep and physical activity). During this session, the pharmacist interviewed the participant, with translation by the CHW if needed, to reconcile the medication list. Lifestyle, cultural factors, and health literacy were also discussed as relevant for each participant. Together, all three collaborated to identify and categorize DTPs.

### Measures

#### Outcome

*Drug Therapy Problems (DTPs).* Determination of DTPs followed guidelines of the Pharmacists’ Patient Care Process set forth by the Joint Commission of Pharmacy Practitioners (Joint Commission on Pharmacy Practitioners, 2014). This is the gold standard approach to the assessment of DTPs and was created by expert review of key source documents on pharmaceutical care and medication therapy management. The classification system has four major categories: appropriateness (is the medication indicated for the condition in this particular patient?), effectiveness (is the medication meeting clinical target?), safety (does the patient have, or is the patient at risk for, adverse drug reactions?), and adherence (is the patient taking the medication as prescribed?). DTPs are assessed and classified in that sequence so that problems associated with the medication itself are not attributed to patient non-adherence. The pharmacist identified DTPs by reviewing the participant’s medication review form that had been filled out by the CHW, reviewing clinical data from the baseline study assessments and discussing pertinent findings with the participant. The pharmacist discussed each medication and clinical condition with the patient, detected any DTPs, determined the appropriate DTP code, and then documented the presence and type of DTP in the pharmacy research record.

#### Predictors of DTPs

*Demographic Characteristics* included age, sex, income, employment status (working vs not) and insurance status (Medicare, Medicaid vs private).

*Understanding.* Participants reported their educational attainment and responded to items regarding their ability to speak, read, and write English, each on a single 4-point scale from ‘not at all’ to ‘very well’. One item asked how often in the past year they experienced difficulty communicating with their healthcare provider because of a language difference using a 5-point scale from ‘never’ to ‘always’ with higher scores indicating greater difficulty. Participants also completed a 10-item true/false/don’t know quiz that asked about what diabetes is, risk factors for developing it, and strategies for prevention.

*Social Determinants.* Social support was measured with four items from the PROMIS test bank (Cella et al., 2007) that tap into domains of instrumental, informational, emotional, and companionship support and one item from the Enriched Social Support Instrument (Mitchell et al., 2003), which taps into love and affection. Responses were on a 5-point scale with higher scores indicating greater social support. Responses were summed. We also assessed marital status (married vs not) and living alone (yes vs no).

*Physical Function.* Self-reported health was measured with one item on 5-point Likert scale ranging from ‘excellent’ to ‘poor’ (Hays, Bjorner, Revicki, Spritzer, & Cella, 2009) and another item regarding change in health status over the past year ranging from ‘much better than a year ago’ to ‘much worse than a year ago’ (Wagner et al., 2013). Participants also reported the presence of pain in the past 7 days with three questions assessing the degree of difficulty doing day-to-day activities due to pain, modeled after the SF-36 items (Ware & Sherbourne, 1992); scores range 0 - 5 with higher scores indicating greater pain and interference.

*Mental Health.* We assessed symptoms of Post-Traumatic Stress Disorder (PTSD) with the 16-item symptom subscale of the Khmer language version of the Harvard Trauma Questionnaire using the standard cutoff of 2.5 (Mollica et al., 1992) to determine likely PTSD. We measured symptoms of depression with the 15-item depression subscale of the Khmer language version of the Hopkins Symptom Checklist and anxiety with its 10-item anxiety subscale using published cutoffs of 1.75 (Mollica et al., 1987) to determine likely depression and anxiety disorder, respectively.

### Data analysis

A multivariable Poisson regression model was run to assess the association between significant patient measures found by bivariate correlations and the total number of DTPs. The incidence rate ratio (exponentiated coefficient) is reported as a measure of effect. The log of the number of medications taken was included in the Poisson model as an offset to account for the fact that more medications lead to greater opportunity for DTPs. Data were available for all variables and thus analyses included all participants; no strategy for handling missing data was needed. Because this was secondary data analysis from a larger study, no power analysis was conducted. Significance was set *a priori* at *p* = 0.05. Data analyses were conducted in SPSS v26.

## Results

### Participants

We screened 540 recruits, 206 recruits were consented, and 188 participants were randomized. Participants randomized to MTM (*n* = 63) are reported in this manuscript; they were an average age of 55.1 years (SD = 8.4) and were mostly female (83%), married (52%), spoke Khmer at home (87%), and were not working (68%). See Table 1 for a detailed description of the sample.

**Table 1.** Demographic characteristics (*n* = 63).

Characteristic	Frequency	Percentage
*Gender*		
Female	52	82.5%
Male	11	17.5
*Age*		
Mean ± SD	55.1 ± 8.4	
*Marital status*		
Married	33	52.4%
Not married	30	47.6
*Household number*		
1	7	11.1%
2	12	19.0
3–4	23	36.5
5+	21	33.3
*Home language*		
Khmer	55	87.3%
English	6	9.5
Other	2	3.2
*Household income*		
Under $20,000	26	41.3%
$20,000–30,000	16	25.4
$31,000–40,000	6	9.5
Over $40,000	6	9.5
Don’t know/Refuse	9	14.4
*Employment status*		
Working	18	28.6%
Not working	43	68.3
Don’t know/Refuse	2	3.2
*Years education*		
Mean ± SD	6.4 ± 5.2	
*Health insurance*		
Medicare	18	28.6%
Medicaid	19	30.2
Private	20	31.7
None/Don’t know	6	9.6

Note: SD, standard deviation.

### Drug therapy and drug therapy problems

Participants took an average of 5.5 (SD = 4.0; range 0-17) medications. The conditions for which they took the most medications were: preventative (e.g. for bone health, mean = 2.0), pain (mean = 1.1), cardiovascular disease (mean = 0.8), mental health (mean = 0.7), asthma (mean = 0.2), and sleep (mean = 0.03). The use of complementary and alternative medications was uncommon; only 3 participants used traditional Khmer medications.

Table 2 shows the number of DTPs classified by problem type. The most common type of DTP was safety (64%) and the average was 1.8 safety DTPs per participant. Almost 45% had at least one appropriateness DTP with an average just under one (mean = 0.9). Nearly 25% had an effectiveness DTP and over 30% had an adherence DTP. Overall, there was an average of 3.7 DTPs per patient with only six participants (10%) experiencing no DTPs.

**Table 2.** Drug therapy problems (*n* = 63).

Problem	Frequency	Percentage
*Appropriateness*		
0	35	55.6%
1	15	23.8
2+	13	20.6
Mean ± SD	0.9 ± 1.4	
*Effectiveness*		
0	48	76.2%
1	11	17.5
2+	4	6.3
Mean ± SD	0.3 ± 0.7	
*Safety*		
0	23	36.5%
1	19	30.2
2+	21	33.3
Mean ± SD	1.8 ± 2.4	
*Adherence*		
0	43	68.3%
1	11	17.5
2+	9	14.3
Mean ± SD	0.8 ± 1.5	
*Total*		
*Mean ± SD*	3.7 ± 3.1	Range: 0–15

Note: SD, standard deviation.

### Correlations

Bivariate correlations between participant factors and number of DTPs by type are shown in Table 3.

**Table 3.** Zero order correlations among participant characteristics and major categories of drug therapy problems.

Measures	Appropriateness problems	Effectiveness problems	Safety problems	Adherence problems	Total problems
*Demographics*					
Age	0.25	0.14	0.07	0.12	0.26*
Sex	0.07	−0.04	−0.13	−0.23	−0.19
Income	−0.05	0.04	−0.21	−0.17	−0.27*
Employment	−0.01	0.01	−0.12	−0.01	−0.10
Insurance	0.30	0.21	0.17	0.26	0.26
*Understanding*					
Speak English	−0.01	−0.06	−0.02	−0.22	−0.14
Read English	0.00	−0.17	−0.14	−0.11	−0.20
Write English	0.10	−0.12	−0.14	−0.17	−0.17
Difficult Communicate	−0.06	0.14	0.11	−0.11	−0.04
Diabetes Quiz	−0.01	−0.06	−0.23	−0.25*	−0.32*
*Social determinants*					
Social Support	0.05	0.17	−0.21	−0.24	−0.22
Married	0.00	0.05	−0.32*	−0.31*	−0.39**
Living Alone	0.07	−0.17	0.05	0.19	−0.13
*Physical function*					
Current Health	−0.01	−0.11	0.22	−0.03	0.21
Change in Health	−0.11	−0.06	−0.37**	0.06	−0.25*
*Mental health*					
Trauma	0.33**	0.13	0.10	−0.14	0.19
Anxiety	0.12	0.02	0.27*	−0.10	0.22
Depression	0.22	−0.11	0.28*	−0.05	0.26*

*p < .05, **p < .01.

### Multivariate associations predicting total number of DTPs

Table 4 shows the results of the Poisson regression models where the variables with significant bivariate correlations were entered in a single model predicting total number of DTPs. When the variables were entered together in a model two associations were significant. Being married reduced the number of drug therapy problems (IRR = 0.70) and being clinically depressed as measured by the Hopkins scale increased the number of problems (IRR = 1.26).

**Table 4.** Multivariate Poisson regression model predicting total number of drug treatment problems.

	Model**^a^**		
Predictors	IRR	95% CI	*P*-value
Age	1.02	1.00–1.04	.078
Income	0.96	0.79–1.15	.622
Diabetes Quiz	0.96	0.90–1.01	.139
Married	0.70	0.50–0.99	.048*
Change in Health	0.97	0.83–1.14	.709
Hopkins Depression	1.26	1.01–1.56	.039*

*p < .05

^a^ Model controls for number of medications.

## Discussion

DTPs were common in this population of Cambodian Americans with depression and high diabetes risk; 90% had at least one DTP, with safety being the most common type. Psychosocial patient characteristics – demographics, understanding, social determinants, physical function and mental health – variously increased risk for safety, appropriateness, effectiveness, and adherence DTPs. In multivariate analysis predicting all types of DTPs combined, being unmarried and having elevated symptoms of depression were risk factors for higher total number of DTPs, even after accounting for number of medications. Pharmacist/CHW teams were key to obtaining sensitive person-level information for a thorough medication review in this vulnerable population.

The main finding from this study is that psychosocial and contextual factors predicted DTPs. Patient characteristics were associated with appropriateness, safety, and adherence, but not effectiveness. Appropriateness refers to a medication’s indication for a particular condition with an acceptable risk/benefit ratio for a particular patient. Examples of appropriateness DTPs are an unnecessary medication or the need for a medication that has not been prescribed (e.g. untreated hypertension). Appropriateness DTPs were related to clinically elevated symptoms of PTSD. Some participants may not have mental health providers, and their primary care providers may not be trained in trauma-informed care. Patients with PTSD symptoms, for their part, may not readily volunteer symptoms of PTSD in part because avoidance of anything associated with the trauma is a key feature of PTSD. These factors may link PTSD symptoms to DTPs.

Safety is the absence of preventable harm to a patient during medication use. Examples of safety DTPs are a dose too high, contraindications, drug interactions, or adverse drug reactions. Safety DTPs were related to four patient characteristics – being unmarried, declining self-reported health over the past year, and clinically significant symptoms of anxiety and depression. Spouses can help monitor for side effects in the patient and serve as collateral informants during healthcare visits. Patients with worsening health status, anxiety and/or depression may receive care from multiple providers and if that care is not carefully coordinated, this can lead to drug interactions.

Adherence is the degree to which the use of medication by the patient corresponds with the prescribed regimen. Examples of adherence DTPs are not filling a prescription, taking the medication in an incorrect way, skipping doses, or taking medications prescribed for other people. Adherence DTPs were related to two patient characteristics – being unmarried and low knowledge of diabetes risk. Being unmarried may leave patients without someone to fill prescriptions or remind them to take their medication. Low knowledge of diabetes in a population of those at high risk for diabetes and its treatment may reflect a general lack of knowledge about chronic disease management. Knowledge of diabetes might improve patient motivation to take medications for chronic conditions on an ongoing basis rather than episodically.

When all significant patient variables were entered into the equation predicting total DTPs, being unmarried and having clinically elevated symptoms of depression were significant risk factors for total DTPs. Some vulnerabilities of being unmarried are discussed above. It should be noted that whereas being unmarried increased DTPs, low social support and living alone were *not* significant predictors of DTPs. It seems that marriage *per se* confers protection against DTPs. Spouses have a longer history with the patient than other members of the social network and so may be better judges of changes to the patient’s functioning.

Clinically elevated symptoms of depression were also associated with a higher number of total DTPs. Low self-worth, feeling helpless and/or hopeless may contribute to a low sense of efficacy about medications and difficulty concentrating may interfere with medication-taking behaviors. It may also be the case that recalcitrant depression triggers more aggressive depression treatment that increases the opportunity for DTPs (Murugappan, Seifert, & Farley, 2020).

DTPs do not occur equitably across populations (Garin et al., 2021; Kaufmann, Stämpfli, Hersberger, & Lampert, 2015; Roth et al., 2011; Sell & Schaefer, 2020). Little is known about DTPs in the Cambodian American population, but the evidence that does exist suggests that they have high rates (Buckley et al., 2019). Direct comparisons with our young (mean age 55.1 years) but psychiatrically vulnerable sample, who took relatively few medications (mean = 5.5) and were recruited from the community are difficult because many DTP prevalence studies investigate older samples taking a high number of medications, data for which come from inpatient hospital records, outpatient clinics, pharmacy registries, or specific disease populations. Also, we analyzed data from a single MTM session, whereas many studies report a total prevalence of DTPs across numerous MTM sessions, in which additional DTPs can emerge over time. Notwithstanding the difficult comparison, our finding of 90% prevalence of any DTP is actually higher than reports from older, sicker samples. For example, DTPs were found in 62% of *n* = 1090 across 300 community pharmacies in Germany with a mean age of 72 years (Sell & Schaefer, 2020), and 45% of *n* = 1602 hospital admissions to medical wards in Europe with a mean age 72 years (Garin et al., 2021).

In addition to the prevalence of any DTP, the number (3.7) and most common type (safety) of DTP that we report here appears higher and potentially more serious than other samples, especially in light of the overall low number of medications taken by our participants. For example, in one study of *n* = 88 primary care patients in Connecticut with a mean age of 51.0 years and 15.7 medications, researchers found an average of 2.3 DTPs per session (Smith, Giuliano, & Starkowski, 2011), with indication the most common. In a study of *n* = 689 patients of an outpatient family medicine residency clinic in Minnesota, with a mean age of 53.6 years and 11.9 medications (MacDonald, Chang, Wei, & Hager, 2018), pharmacists detected 4.2 DTPs per patient, with effectiveness the most common. In another study of *n* = 9068 receiving pharmacy services from a large healthcare organization in Minnesota, in which patients ranged in age from 21 to 102 and were taking on average 12.4 medications (de Oliveira, Brummel, & Miller, 2020), pharmacists detected 1.2 DTPs per session, with effectiveness being the most common.

Two studies provide particularly informative comparisons. First, one study, like ours, found that safety was the most common type of DTP (Levine et al., 2021). It was a study of *n* = 105 people aged >65 receiving home-based team care for dementia, delirium or depression (3 Ds) in Connecticut, in which researchers focused on medications treating or exacerbating cognition (Levine et al., 2021). A second study found a very high rate of DTPs. Among *n* = 200 aged in their 70’s requiring eldercare in the U.S. (Roth et al., 2011), the DTP rate was 100%. Thus, although the mean age of our sample was 55.1 years and they were not recruited from a particular clinical service, in some regards, the DTPs in our population resemble DTPs among individuals who require homecare.

### Clinical implications

Our findings illustrate that risk factors for DTPs extend beyond the characteristics of the medication regimen to include the patient’s own psychosocial and contextual factors. In fact, patient factors predicted DTPs above and beyond polypharmacy. We contend that prescribers and pharmacists would better manage medications by adopting a holistic view of patients and attending to psychosocial attributes.

We assert that a team approach is necessary for coordinated care of patients with complex medical, psychiatric, and social needs. One study concluded that DTPs were less common when multidisciplinary teams were providing input on care (Kondo et al., 2015). We agree with Wheat et al. (2020) and Segal et al. (2020), who promote teams consisting of CHWs and pharmacists. CHW and pharmacist teams can engage patients in their own care, which is associated with lower DTPs (Eassey et al., 2016; Niriayo et al., 2018). CHWs play a unique role by engaging medically underserved communities and overcoming structural, linguistic, and cultural barriers to care (Cherrington et al., 2008). As typically bilingual, bicultural and respected members of the community, CHWs bridge the community and healthcare establishment.

## Limitations

Limitations of this study include a small and unique sample that may not generalize to other groups. The secondary analysis was cross-sectional, so temporal precedence and causality cannot be inferred. Strengths include a high risk, hard to reach and understudied population; use of bilingual, bicultural data collectors; investigation of multiple potential patient-level determinants of DTPs; and statistical control for a number of medications. Future studies should follow larger groups longitudinally to determine, for example, whether DTPs are the cause or consequence of depressive symptoms.

## Conclusion

We found that DTPs are common in this population and that being unmarried and having depression increases odds for DTPs. The World Health Organization promotes a prescribing culture that prioritizes safety and quality and underscores the importance of a multi-professional team. As the migrant crisis continues across the globe and their healthcare remains a largely unmet need, data from long-settled migrant groups like that reported here are informative. Those who are unmarried and who have symptoms of depression should be prioritized for medication therapy management.

## Supplementary Material

Supplemental Material
